# Enhancement of anti-OVA IgG2c production *in vivo* by enalapril

**DOI:** 10.1590/1414-431X20165215

**Published:** 2016-07-11

**Authors:** L.C. Almeida, L.S. Muraro, D.A. Albuquerque

**Affiliations:** 1 Departamento de Biomedicina, Centro Universitário Cândido Rondon, Cuiabá, MT, Brasil; 2 Departamento de Patologia Clínica e Parasitologia, Faculdade de Medicina Veterinária, Universidade de Cuiabá, Cuiabá, MT, Brasil; 3 Departamento de Ciências Básicas em Saúde, Faculdade de Medicina, Universidade Federal de Mato Grosso, Cuiabá, MT, Brasil

**Keywords:** ACE inhibitors, Enalapril, Humoral response, IgG2c antibodies

## Abstract

Angiotensin-converting enzyme (ACE) inhibitors have non-hemodynamic, pleiotropic effects on the immune response. The effects of ACE inhibitors on the production of cytokines and T-cell functions are well established. However, little is known on the effects of these medicines on humoral response to foreign antigens. In this study, we investigated the effect of enalapril treatment on ovalbumin (OVA)-specific IgG1 and IgG2c production in mice determined by ELISA. Two groups of 8-week-old C57BL/6 females mice (3–4/group) were subcutaneously immunized with OVA (10 μg/animal) in presence of Alhydrogel (1 mg/mouse) and boosted at day 21. The mice were treated with enalapril (5 mg/kg daily, *po*) or were left without treatment for one month. The animals were bled from the orbital plexus on days 0, 7, 14, 21, and 28 after the first immunization and the sera were stored at –20°C until usage. OVA-specific serum IgG1 and IgG2c were determined by ELISA using serum from each individual animal. The results showed that enalapril significantly increased anti-OVA serum IgG2c in the secondary response without affecting IgG1 synthesis. These data expand our understanding on the properties of enalapril on the immune response, including antibody production.

## Introduction

The antibody response to proteins depends on simultaneous activation of Ag-specific cognate B and T cells. Additionally, the antibody isotope, like IgG2a/IgG2c and IgG1 produced by B cells in response to T-dependent immunogens, is driven by cytokines produced by Th1 and Th2 lymphocytes, respectively (1–3). T-cell polarization into either Th1 or Th2 profile is influenced by several endogen signals, including cytokines produced by Ag-presenting cells during the onset of T-cell response. It is also well established that exogen agents such as adjuvants and some medicines are involved in shaping the following immune response, and thus have a major impact on the profile of the subsequent T-cell response. In this regard, a large body of clinical and experimental studies has established that angiotensin-converting enzyme (ACE) inhibitors, such as enalapril, captopril, and lisinopril have pleiotropic, non-hemodynamic properties on T-cell response by inducing cytokine synthesis (4,5). Accordingly, we have demonstrated that captopril, an ACE inhibitor with a thiol group, inhibits the production of IL-10 and IL-4 without affecting IL-5, IFN-γ, and IL-2 synthesis in lupus mice (6). In agreement with our findings, it was recently reported that captopril reduced the production of TNF-α, IL-1α, IL-10, IL-12, and IL-18 by LPS-stimulated dendritic cells (7). In a previous study, we showed that enalapril, an ACE inhibitor without a thiol group, significantly increased the number of CD4^+^CD103^+^CD25-negative T cells in the spleen of normal Balb/c mice together with the increasing production of IL-10 (8). Moreover, it was recently shown that enalapril induced an expansion of T cells and re-polarization of macrophages towards a M1-like state in kidneys of diabetic mice (9).

So far, most of the studies on immune-mediated properties of ACE inhibitors have emphasized their effects on cytokine production and T cell activation (4–9). Little attention, however, has been paid to possible immune-modulatory roles of ACE inhibitors on antibody synthesis. In this regard, data from two clinical studies showed that patients treated with captopril or lisinopril developed IgM anti-double-stranded DNA and IgG anti-(H 2A-H 2B)-DNA antibodies, respectively (10,11). However, using the same pharmacological approach, we showed that captopril does not affect IgG anti-dsDNA antibodies in lupus-prone BWF1 mice (6). Reinforcing our data, it has been shown that captopril does not alter the production of myosin-specific antibodies in antigen-immunized mice (12 [Bibr B13] [Bibr B14] [Bibr B15]). Based on our and other authors' findings (10–12), it could be hypothesized that, at least regarding captopril effects on autoantibody production, data from clinical and experimental studies are contradictory.

To extend our overall comprehension on the effects of ACE inhibitors on antibody production, we sought to analyze whether the widely used ACE inhibitor enalapril would interfere with anti-ovalbumin (OVA) humoral response in mice. Enalapril was chosen as the ACE inhibitor model because it regulates cytokine production and, as far as we know, there is no data in the literature on the effect of this ACE inhibitor on humoral response to foreign antigens in pre-clinical models. In the present work, we have investigated the effect of enalapril on the humoral response of C57BL/6 mice immunized with EndoFit OVA in the presence of Alhydrogel, as adjuvant. Our results showed that enalapril significantly enhanced anti-OVA serum IgG2c without any apparent effect on OVA-specific IgG1.

## Material and Methods

### Animals

Fourteen 8-week-old C57BL/6 female mice used in this study were purchased from CEMIB, UNICAMP, Campinas, SP, Brazil. The animals were kept in micro-isolators and all experiments were performed according to the Universidade Federal de Mato Grosso institutional ethical guidelines on the use of animals in research (# 23108.039341/12-4).

### Antigen and adjuvant

EndoFit OVA and Alhydrogel (Al) were used as antigen and adjuvant, respectively (InvivoGen, USA).

### Immunization

Mice were subcutaneously immunized with OVA (10 µg) in the presence of Al (1 mg) in a total volume of 0.1 mL per animal. Twenty-one days after priming, all animals received a booster under the same condition of the first immunization. Blood samples were collected from each individual mouse on days 0, 7, 14, 21, and 28 after primary immunization and serum was kept at –20°C until used.

### Treatment

OVA-immunized mice were treated with enalapril or were left without treatment (3–4 animals/group). Enalapril (Hexal, Brazil) was dissolved in drinking water at a concentration of 0.02 mg/mL and replaced every 24 h. The daily dosage for enalapril was 5 mg/kg body weight, assuming a daily fluid intake of 5 mL per mouse. This dose of enalapril is within the usual therapeutic ranges used in mice (8,).

### ELISA assay

OVA-specific IgG1 was assayed by standard ELISA procedures. In short, polystyrene plates (Maxsorp, Denmark) were coated with OVA (10 μg/mL) overnight at 4°C, washed with PBS containing 0.05% Tween-20, blocked with 1%-BSA for 1 h at room temperature, and incubated with serial dilutions of each mouse antiserum starting at 1/100. After overnight incubation at 4°C, the plates were washed, incubated for 1 h at 37°C with rabbit anti-mouse IgG1 conjugated to peroxidase (Zymed, USA), washed and developed by addition of H_2_O_2_ and orthophenylenediamine. The reaction was stopped by addition of H_2_SO_4_, the absorbance at 492 nm was read using a microplate reader (Thermo Plate-Reader, NM, X model, China) and the results were reported as the mean absorbance ± SD. Anti-OVA IgG2c was assayed following manufacturer's instructions (eBioscience, USA). IgG2c concentration was determined by extrapolation from the standard curve. Minimum level of detection for IgG2c is 0.39 ng/mL.

### Measurement of serum IFN-γ

Serum IFN-γ levels were determined by ELISA, using monoclonal antibody pairs and recombinant cytokines (eBioscience, USA), as previously described (6).

### Statistical analysis

The results were analyzed using one-way analysis of variance (ANOVA) followed by the Tukey test. P≤0.05 was considered to be statistically significant.

## Results

**Figure 1:**
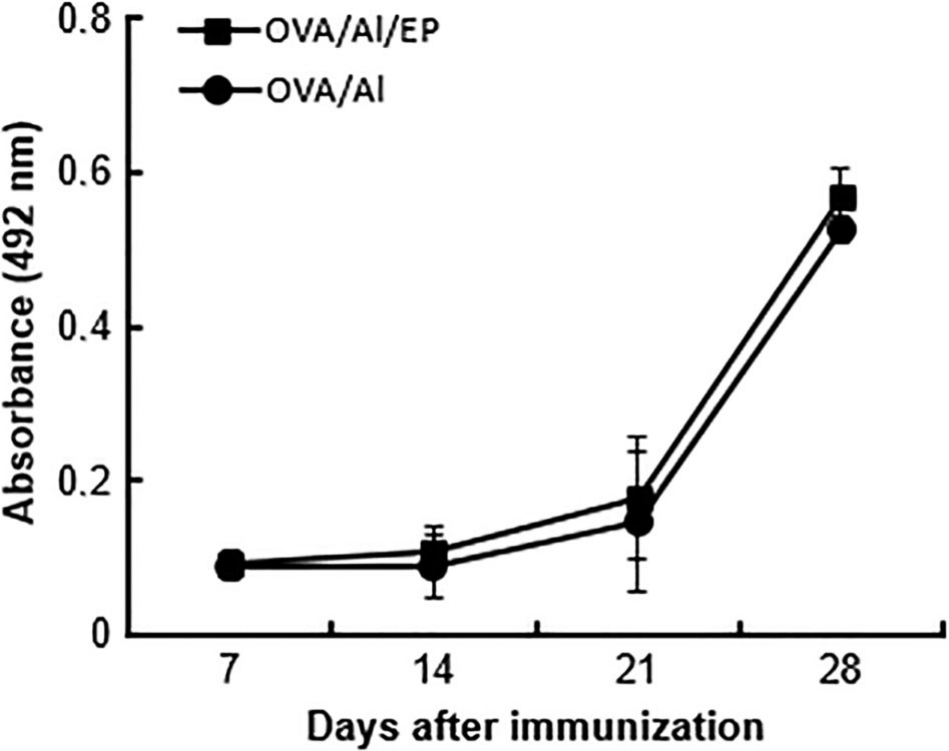
Evaluation of the effect of enalapril (EP) on ovalbumin (OVA)-specific IgG1. Two groups of mice (3-4/group) were subcutaneously immunized with OVA/Alhydrogel (Al) and boosted at day 21. The mice were orally treated with enalapril or were left without treatment. OVA-specific IgG1 was determined by ELISA using 1:400 diluted serum. Data are reported as means±SD and represent 2 independent experiments.

To investigate whether enalapril would regulate antibody response against OVA, mice injected with EndoFit OVA plus Al were treated with enalapril for 4 weeks. For comparative purposes, control animals were immunized with OVA/Al and were left without enalapril treatment. Data reported in Figure 1 show that kinetics of OVA-specific IgG1 response exhibited a classic pattern, with anti-OVA serum IgG1 levels increasing with time and after the booster. These results were not affected by enalapril treatment. In a similar study, it was shown that captopril, an ACE inhibitor with a thiol group, does not affect OVA-induced IgG responses in mice immunized with this antigen in complete Freund's adjuvant (12). Taken together, these data add novel information on the lack of effects of ACE inhibitors on anti-OVA IgG1 response in the mouse model. It should be noted that mice immunized with EndoFit OVA plus Al and treated or not with enalapril did not exhibit OVA-specific IgG1, on days 7 and 14 after priming (data not shown). The lack of OVA-specific serum IgG1 at early primary response may be explained by the lack of LPS in EndoFit OVA, as TLR ligation on B cells may be necessary for T-dependent antibody responses (16).

**Figure 2:**
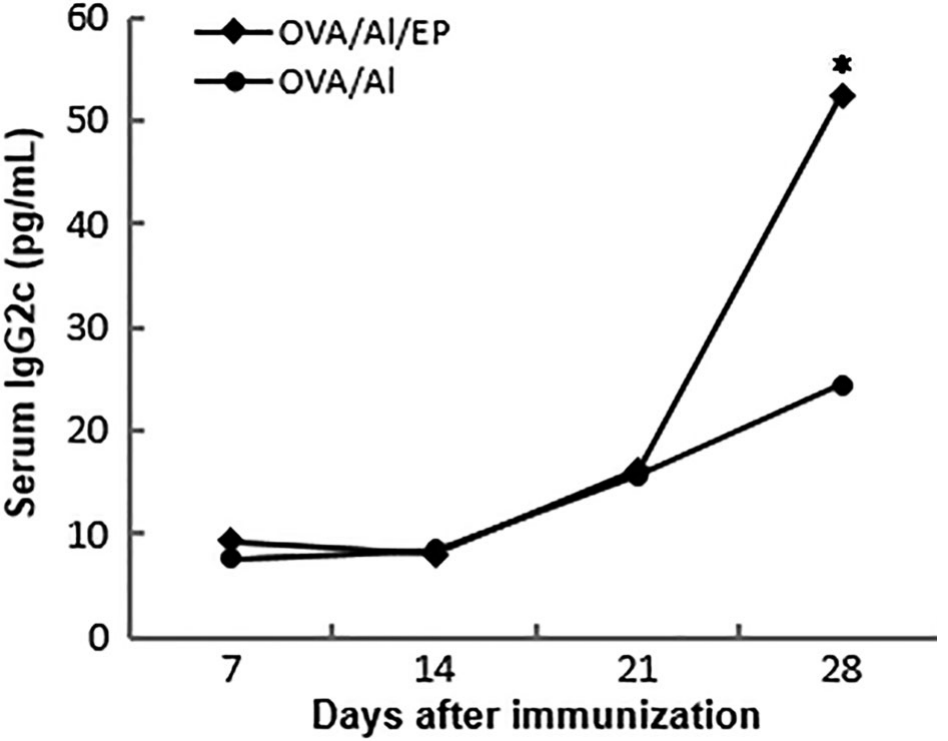
Levels of anti-ovalbumin (OVA) IgG2c during the study period. Two groups of mice (3-4/group) were subcutaneously immunized with OVA/Alhydrogel (Al) and boosted at day 21. The mice were orally treated with enalapril (EP) or were left without treatment. OVA-specific IgG2c levels were determined by ELISA using 1:1000 diluted serum. Data are reported as means and represent 2 independent experiments. *P=0.0006, one-way ANOVA followed by the Tukey's test.

To extend our overall understanding on the effects of enalapril on humoral response, we also evaluated serum anti-OVA IgG2c. As shown in Figure 2, the levels of OVA-specific IgG2c observed in the primary response (7, 14, and 21 days after the first immunization) were similar in both enalapril-treated and in enalapril-untreated mice. However, at the 28th day after the booster, OVA-specific IgG2c levels were significantly higher (P=0.0006) in enalapril-treated mice in comparison with untreated animals (Figure 2). These data suggest that enalapril up-regulates systemic IgG2c production in late humoral response to a foreign T-dependent antigen. As expected, no OVA-specific antibodies (IG1 and IgG2c) were detected before priming (data not shown).

## Discussion

In this study, we evaluated the effect of enalapril, an ACE inhibitor without a thiol group, on humoral response in C57Bl/6 mice immunized with EndoFit OVA plus Al, as adjuvant. The data showed that enalapril did not affect anti-OVA IgG1 response but it significantly increased OVA-specific IgG2c in secondary response (7 days after the booster). Our findings that enalapril does not alter OVA-specific IgG1 production are in line with those showing that anti-OVA IgG1 response was not affected by captopril (12). However, in our design, mice that were given enalapril exhibited significantly higher IgG2c levels compared to enalapril-untreated animals (Figure 2). As far as we know, our data on the increase of IgG2c response by enalapril are novel and are supportive of the ACE inhibitors' role on humoral response to foreign antigens in mice. As switching to IgG2c is largely dependent on signals from IFN-γ (1,2), we wondered whether the increase in IgG2c production by enalapril was due to the increase in circulating IFN-γ. We found that enalapril had no effect on serum IFN-γ levels (data not shown). Although circulating IFN-γ levels were not affected in enalapril-treated mice, a possible inhibition of Th2 response by enalapril could not be ruled out, as we only measured IFN-γ by ELISA, by which the minimum level of detection for IFN-γ is 15 pg/mL. Our hypothesis that enalapril may inhibit local Th2 response supports recently reported results showing that enalapril re-polarized macrophages towards a M1-like state in kidneys of diabetic mice (9). Whether the enhancement of IgG2c production verified in our experimental design is related to the inhibition of local Th2 response by enalapril needs to be further evaluated.

The rational for studying the effect of ACE inhibitors on humoral response relies on the fact that enalapril and captopril are included in the list of medicines implicated in drug-induced lupus (11). As ACE inhibitors are widely used to treat hypertensive patients, and primary hypertension is a chronic condition, one would be concerned with the effects of these medicines in shifting patient's Th response to the Th1 profile, which might favor the development of autoimmune diseases governed by Th1 response.

Our results suggest that systemic inhibition of angiotensin II by enalapril up-regulates IgG2c production without increasing serum IFN-γ levels. Our results have important implications in the understanding of the regulatory effects of ACE inhibitors on humoral response, mainly opening a new perspective of research on the role of these medicines on humoral response to foreign T-dependent antigens.

## References

[1] Bossie A, Vitetta ES (1991). IFN-gamma enhances secretion of IgG2a from IgG2a-committed LPS-stimulated murine B cells: implications for the role of IFN-gamma in class switching. Cell Immunol.

[B02] Snapper CM, Peschel C, Paul WE (1988). IFN-gamma stimulates IgG2a secretion by murine B cells stimulated with bacterial lipopolysaccharide. J Immunol.

[3] Snapper CM, Finkelman FD, Paul WE (1988). Differential regulation of IgG1 and IgE synthesis by interleukin 4. J Exp Med.

[4] Gullestad L, Aukrust P, Ueland T, Espevik T, Yee G, Vagelos R (1999). Effect of high- versus low-dose angiotensin converting enzyme inhibition on cytokine levels in chronic heart failure. J Am Coll Cardiol.

[B05] Platten M, Youssef S, Hur EM, Ho PP, Han MH, Lanz TV (2009). Blocking angiotensin-converting enzyme induces potent regulatory T cells and modulates TH1- and TH17-mediated autoimmunity. Proc Natl Acad Sci U S A.

[B06] De Albuquerque DA, Saxena V, Adams DE, Boivin GP, Brunner HI, Witte DP (2004). An ACE inhibitor reduces Th2 cytokines and TGF-beta1 and TGF-beta2 isoforms in murine lupus nephritis. Kidney Int.

[B07] Lapteva N, Ide K, Nieda M, Ando Y, Hatta-Ohashi Y, Minami M (2002). Activation and suppression of renin-angiotensin system in human dendritic cells. Biochem Biophys Res Commun.

[B08] Albuquerque D, Nihei J, Cardillo F, Singh R (2010). The ACE inhibitors enalapril and captopril modulate cytokine responses in Balb/c and C57Bl/6 normal mice and increase CD4(+)CD103(+)CD25(negative) splenic T cell numbers. Cell Immunol.

[9] Cucak H, Nielsen FL, Hojgaard PM, Rosendahl A (2015). Enalapril treatment increases T cell number and promotes polarization towards M1-like macrophages locally in diabetic nephropathy. Int Immunopharmacol.

[10] Kallenberg CG, Hoorntje SJ, Smit AJ, Weening JJ, Donker AJ, Hoedemaeker PH (1982). Antinuclear and antinative DNA antibodies during captopril treatment. Acta Med Scand.

[B11] Carter JD, Valeriano-Marcet J, Kanik KS, Vasey FB (2001). Antinuclear antibody-negative, drug-induced lupus caused by lisinopril. South Med J.

[12] Godsel LM, Leon JS, Wang K, Fornek JL, Molteni A, Engman DM (2003). Captopril prevents experimental autoimmune myocarditis. J Immunol.

[B13] Tarkowski A, Carlsten H, Herlitz H, Westberg G (1990). Differential effects of captopril and enalapril, two angiotensin converting enzyme inhibitors, on immune reactivity in experimental lupus disease. Agents Actions.

[B14] Herlitz H, Svalander C, Tarkowski A, Westberg G (1988). Effect of captopril on murine systemic lupus erythematosus disease. J Hypertens Suppl.

[B15] Perez De Lema G, De Wit C, Cohen CD, Nieto E, Molina A, Banas B (2003). Angiotensin inhibition reduces glomerular damage and renal chemokine expression in MRL/lpr mice. J Pharmacol Exp Ther.

[16] Pasare C, Medzhitov R (2005). Control of B-cell responses by Toll-like receptors. Nature.

